# Push Notifications Reduce Emergency Department Response Times to Prehospital ST-segment Elevation Myocardial Infarction

**DOI:** 10.5811/westjem.2018.12.40375

**Published:** 2019-02-11

**Authors:** Mathew Goebel, Joseph Bledsoe

**Affiliations:** * University of California San Diego School of Medicine, Department of Emergency Medicine, San Diego, California; † Intermountain Medical Center, Department of Emergency Medicine, Murray, Utah

## Abstract

**Introduction:**

Prehospital acquisition of electrocardiograms (ECG) has been consistently associated with reduced door-to-balloon times in the treatment of ST-segment myocardial infarction (STEMI). There is little evidence establishing best hospital practices once the ECG has been received by the emergency department (ED). This study evaluates the use of a push notification system to reduce delays in cardiac catheterization lab (CCL) activation for prehospital STEMI.

**Methods:**

In this prospective before-and-after study, we collected prehospital ECGs with computer interpretation of STEMI from May 2012 to October 2013. Push notifications were implemented June 1, 2013. During the study period, we collected timestamps of when the prehospital ECG was received (email timestamp of receiving account), CCL team activation (timestamp in paging system), and patient arrival (timestamp in ED tracking board). When prehospital ECGs were received in the ED, an audible alert was played via the Vocera WiFi communication system, notifying nursing staff that an ECG was available for physician interpretation. We compared the time from receiving the ECG to activation of the CCL before and after the audible notification was implemented.

**Results:**

Of the 56 cases received, we included 45 in our analysis (20 cases with pre-arrival CCL activation and 25 with post-arrival activation). For the pre-arrival group, the interval from ECG received to CCL activation prior to implementation was 9.1 minutes with a standard deviation (SD) of 5.7 minutes. After implementation, the interval was reduced to 3.33 minutes with a SD of 1.63 minutes. Delay was decreased by 5.8 minutes (p < 0.01). Post-implementation activation times were more consistent, demonstrated by a decrease in SD from 5.75 to 1.63 min (p < 0.01). For patients with CCL activation after arrival, there was no significant change in mean delay after implementation.

**Conclusion:**

In this small, single-center observational study, we demonstrated that the use of push notifications to ED staff alerting that a prehospital STEMI ECG was received correlated with a small reduction in, and increased consistency of, ED CCL activation.

## INTRODUCTION

Prehospital acquisition of electrocardiograms (ECG) has been consistently associated with reductions in door-to-balloon (D2B) times for the treatment of ST-segment myocardial infarction (STEMI) ranging from 15 to 50 minutes.^1^–^10^ The 2015 American Heart Association Guidelines for Emergency Cardiovascular Care made early acquisition of prehospital ECG a Class I recommendation.^11^,^12^ Many emergency medical services (EMS) systems require transmission of the ECG for physician interpretation prior to cardiac catheterization lab (CCL) activation. There is no evidence establishing best practice after the ECG has been received at the hospital. Delays to physician interpretation can occur if test results are misplaced, forgotten, or overlooked in a busy emergency department (ED). We evaluated the use of a push notification system to reduce delays in CCL activation for prehospital STEMI patients.

## METHODS

This was a before-and-after comparison study conducted at a single, urban, academic center in Salt Lake City, Utah, with an annual census of 88,000 patient encounters. The receiving facility ED has 67 beds in three zones, with a minimum of double attending physician coverage 24 hours per day. Patients arrive from multiple EMS agencies in the region. Prehospital medical response and transports are either entirely fire department based, or fire department first response with third service ambulance contracted for patient transport. There are six STEMI receiving centers in the county, and suspected STEMI patients are transported by paramedics to the closest facility based on distance and knowledge of local traffic patterns.

**Figure 1:**
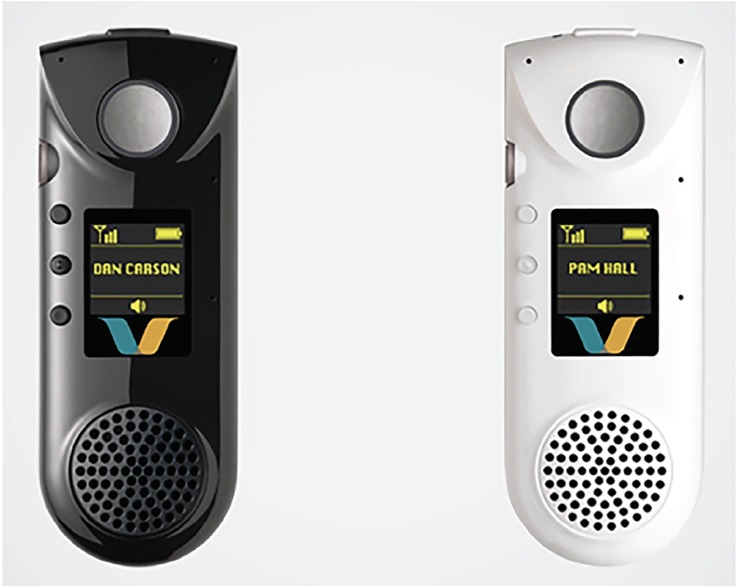
Vocera WiFi communication badge.

Prehospital 12-lead ECGs are transmitted via email attachment using the ambulance’s cardiac monitor. This is at the paramedic’s discretion based on his or her own ECG interpretation or the computer interpretation of the ECG. Prior to implementation of push notifications, ED staff would only periodically check whether prehospital ECGs had arrived. As a result, most patients (even those who had an ECG available prior to arrival) had a 12-lead ECG acquired on hospital equipment upon arrival in the ED. The ECG was interpreted at bedside by the treating emergency physician (EP) who made the decision whether to activate the STEMI protocol. The ED charge nurse then called the “STEMI nurse” via the Vocera WiFi communication system (Vocera Inc., San Jose, California; Figure 1), who served as a single point of contact for CCL activation. The interventional cardiologist then arrived in the ED to assess the patient while the CCL team prepared for the procedure. Interventional cardiologists had the option to over-read the EP’s interpretation before proceeding with the procedure, but this was left to provider preference.

**Population Health Research Capsule**

What do we already know about this issue?

*ST-elevation myocardial infarction (STEMI) is a time sensitive diagnosis that has been shown to benefit from pre-arrival cardiac catheterization lab activation*.

What was the research question?

Do push notifications indicating a pre-hospital electrocardiogram has been received in the emergency department (ED) reduce the time it takes the ED to activate the cardiac catheterization lab?

What was the major finding of the study?

*Push notifications were associated with a reduction in the time ED staff took to activate the cardiac catheterization lab. Additionally, times were more consistent*.

How does this improve population health?

*Faster, more consistent cardiac catheterization activation for patients experiencing STEMI has been associated with improved mortality and possibly improved morbidity*.

**Figure 2:**
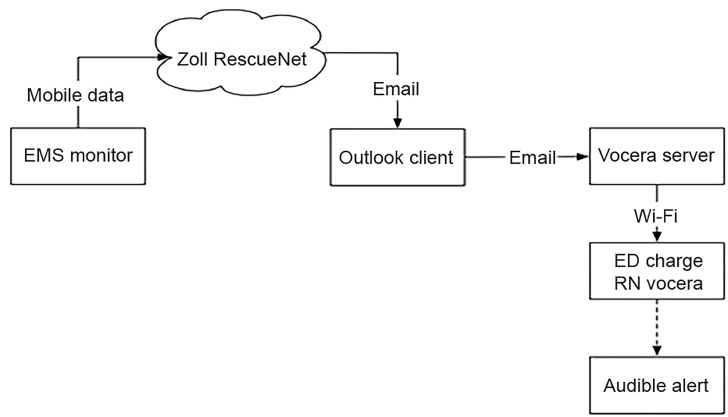
Diagram of a push notification system used to alert ED staff to the incoming transmission of a prehospital electrocardiogram. *EMS*, emergency medical services; *ED*, emergency department; *RN*, registered nurse.

After implementation of push notifications, when prehospital ECGs were received in the ED an audible alert was played to the ED charge nurse via the text-to-speech function of the Vocera WiFi communication system saying, “ECG received, ECG received” (Figure 2). This notified nursing staff that an ECG was available for physician interpretation. The prehospital ECG was shown to an EP, and the same procedure for activating STEMI protocol was followed. If the CCL team indicated they were ready for the patient prior to his or her arrival, the patient was briefly assessed for stability by an EP on the ambulance gurney without being placed in a room and then taken directly to the CCL. If the CCL had not notified the ED they were ready for the patient or the patient was unstable, the patient was placed in an ED room and received any necessary stabilizing treatment until the CCL was ready.

We included adult patients (age ≥ 18 years) arriving from the field with a prehospital ECG consistent with STEMI received prior to patient arrival in the ED – ie, those who could have the CCL activated before arrival in the ED. For the before period, all prehospital ECGs received from the time ECG transmission was implemented until push notifications were implemented (from May 1, 2012, through May 30, 2013) were collected as a historical cohort and screened for enrollment. For the after period, all prehospital ECGs received after implementation were prospectively collected and screened (June 1, 2013 through September 31, 2013). All adult ECGs with a computer interpretation of STEMI were recorded in the data set. We excluded minors (age <18 years), inter-facility transfer patients, patients with ECGs that were transmitted to our facility in error, and ECGs that could not be matched to an ED patient. During the study period, we collected timestamps when the ECG was received, when the CCL team was activated, and when the patient arrived in the ED.

**Figure 3:**
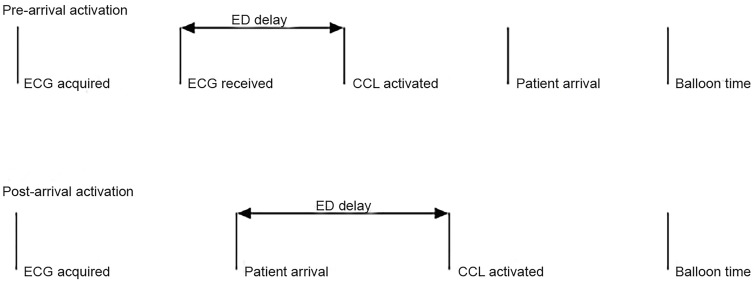
Timestamps used in the study to calculate emergency department delay. *ED*, emergency department; *ECG*, electrocardiogram; *CCL*, cardiac catheterization laboratory.

We calculated time intervals in decimal minutes. For patients where CCL activation occurred prior to ED arrival, “ED delay” was calculated as the time between when the prehospital ECG was received and the CCL was activated. For patients where CCL activation occurred after ED arrival, “ED delay” was calculated as the interval from ED arrival to CCL activation (Figure 3) under the assumption that an EP either did not see the prehospital ECG until the patient’s arrival and wanted an ED-performed ECG, or that the EP wanted to examine the patient personally. These time intervals were treated as continuous data. Because a variety of factors outside the ED’s control affect CCL readiness, we used “ED delay” as our primary endpoint rather than D2B time. We believe this most accurately reflects the portion of D2B time for which the ED has influence. D2B was recorded as a secondary outcome.

We performed statistical analysis in SPSS Statistics (version 24; SPSS Inc., Chicago, Illinois). ED delay was compared using a Wilcox signed-rank test due to the non-parametric distribution of the data. We compared standard deviation (SD) of ED delay using a Mann-Whitney U test, also because of the non-parametric data distribution. D2B times were compared using an independent sample t-test because these data were normally distributed. Continuous demographic data (age) was compared using an independent sample t-test. We compared categorical demographic data (gender, race, risk factors, mortality) using Fisher’s exact test. The proportion of cases activated prior to arrival was compared using Fisher’s exact test. We obtained consent and privacy waivers from the Intermountain Healthcare Institutional Review Board, project # 1050432.

## RESULTS

We collected 56 cases during the study period. Two were excluded as inter-facility transports, two were confirmed as received by our facility in error, and seven ECGs could not be matched to a patient arriving in our ED. The remaining 45 cases included in the analysis represent 43 unique patients. Two patients’ medical record numbers could not be matched when we later performed a query for patient demographics, likely due to a typographic error in the original data entry. Patient demographics are summarized in Table 1.

**Figure 4:**
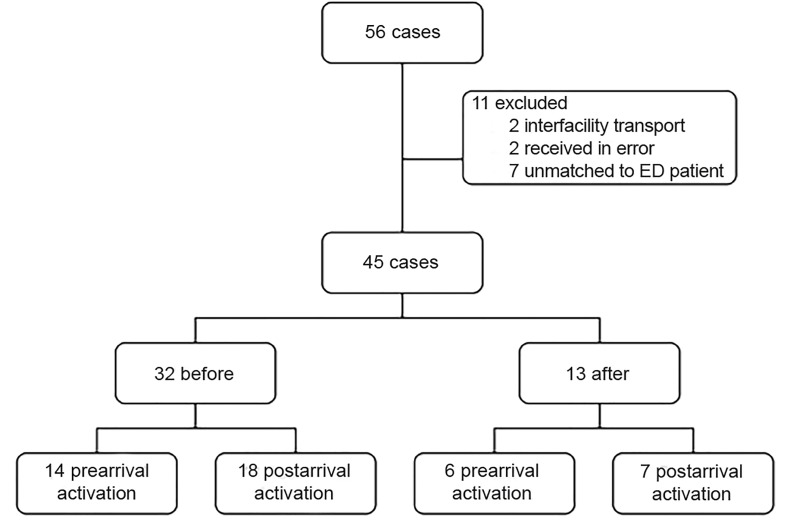
Breakdown of cases collected before and after implementation of push notifications. ED, emergency department.

Of these 45 cases, 32 were received before implementation of the push notification system and 13 were received after. Of the 32 “before” cases, 14 resulted in pre-arrival CCL activation. Of the 13 “after” cases, six resulted in pre-arrival CCL activation. In total, 20 cases were activated prior to the patient arriving in the ED and could be used for analysis of the intervention effect (Figure 4). Every pre-arrival activation continued on to the CCL and received an intervention.

The characteristics of ED delay before and after push notifications are summarized in Table 2. Before push notifications, the mean ED delay for pre-arrival activation was 9.13 minutes (SD 5.75 minutes) and median delay was 6.27 minutes. After implementation, the mean delay was 3.33 minutes (SD 1.63 minutes) and median delay was 3.00 minutes (p<0.01). Observed power for this difference was 82%. Times were also more consistent, demonstrated by a decrease in SD of 4.22 minutes (p < 0.01). For post-arrival patients, there was no significant change in mean; 2.5 minutes before vs 5.3 minutes after (p = 0.55), SD 3.99 before vs 8.57 after (p= 0.44), or median; 1.00 before vs 1.50 after (p = 0.55). There was no significant difference in the rate of pre-arrival activation (p = 1.00). There was a non-significant trend toward a reduction in D2B times for both pre-arrival (57.00 before vs 48.67 after, p = 0.25) and post-arrival activation groups (51.50 before vs 44.00 after, p = 0.14). In post-arrival activation cases, there were no significant differences in ED delay or D2B.

## DISCUSSION

Prehospital ECG transmission has been shown to reduce D2B times and increase the number of cases receiving treatment within 90 minutes. Accordingly, many guidelines for field operations encourage ECG transmission, but no best practices or guidelines examine the hospital’s role in reducing D2B with prehospital ECG transmission – namely workflow – once the ECG is received. This study is novel in that it examines the effect of technology on ED workflow and CCL activation times for prehospital STEMI activations. We demonstrated a small but consistent reduction in ED delay that suggests push notifications may have a role in optimizing ED workflow for prehospital STEMI patients. Our observed reduction may not be clinically significant, but other facilities that currently have longer ED delay may see a larger effect size when implementing this intervention.

We found a non-significant trend toward improvement in D2B times for both groups after implementing push notifications. There are a number of factors outside the ED’s control that affect CCL readiness, such as the time of day and procedures already in progress. Either of these factors, which were not controlled for, may explain why ED delay improved significantly but D2B did not. Additionally, we found no difference in the fraction of prehospital ECGs activated prior to patient arrival. Facilities that have a lower pre-arrival CCL activation rate could see a significant effect when implementing this intervention. We saw several cases (nine) where a diagnostic ECG was received prior to patient arrival, but the CCL was not activated. These patients all continued on to CCL, but without the benefit of the pre-arrival activation they were eligible to receive. This may have occurred as a result of physician preference or other factors affecting the availability of ED staff to get the ECG read in a timely fashion.

There may be a variety of unintended consequences to implementing a push notification system. EPs are already interrupted at a staggering frequency during their shifts.^13^–^16^ This notification process creates an additional source of interruptions for providers at all levels. The system also relies on the availability of other ED staff such as a charge nurse for the system to succeed. Having staff respond to push notifications may draw time and attention from other patient-care tasks with unintended ramifications. Alarm fatigue is also an issue. It is possible this system’s success was due to its novelty and that over time staff could become less responsive. We chose to use the Vocera system because it was an existing technology at our facility and required no additional cost to integrate with our notification system. It is possible that other systems for notification such as text paging, alert lights, or computer pop-ups could have a similar effect depending on what another facility has available.

## LIMITATIONS

This study is limited by its small sample size, single-site design, and use of a convenience sample. We only described the experience of our facility implementing one type of push notification. While we demonstrated limited benefit, it is difficult to generalize this to facilities whose STEMI processes differ from ours. Additionally, only prehospital ECGs with a computer interpretation of STEMI were collected. Any computer false negatives would have been missed, as well as any tracings that failed transmission for any technical reasons (poor wireless connectivity, monitor error, Bluetooth connectivity issue, etc.).

While the various agencies in our EMS system use different brands of cardiac monitors for acquiring 12-lead ECGs, there is significant heterogeneity in the test characteristics of computerized STEMI diagnosis between brands.^17^–^21^ Transmission of the ECG was at the discretion of the treating paramedic. Further, differences in protocols between EMS agencies could also have affected the decision to transmit the ECG. These sources of variance likely affected the number of cases we received. We began collecting data when ECG transmission was first implemented in our region. Although EMS agencies used their existing cardiac monitors, difficulties with the initial implementation may have contributed to our small sample size.

Our goal, however, was to look at effects after the ECG was received in the ED, which involved EP interpretation regardless of the reason for transmission. Thus, while paramedic discretion, computer algorithm accuracy, and differences in EMS protocols may have affected our sample size, it would not systematically bias our measured metric of time to CCL activation, as hospital providers were effectively blinded to these differences.

These results relied on nursing staff to get ECGs to a physician for interpretation and may not be externally valid for facilities that do not use EPs for ECG interpretation, or that use other technologies to send ECG results directly to physicians. If the top priority were solely speed of interpretation, relying on paramedic interpretation of STEMI would likely be fastest. However, this could come at the cost of increased false positives. As best practice evidence is lacking, the decision regarding who interprets the ECG is often made on a local level as a result of interdepartmental consensus.

Additionally, we did not examine downstream effects such as D2B times. While the use of prehospital ECGs has consistently been associated with reductions in D2B and increased proportion of cases receiving intervention within 90 minutes, previous research has failed to show an improvement in mortality when D2B time is reduced to less than 90 minutes.^22^ Thus, the significance of any reduction in D2B depends on a facility’s D2B characteristics prior to any process improvement. It should be noted that while there is no demonstrated mortality benefit of D2B below the 90-minute mark, it is possible there is a morbidity benefit in patient-oriented measures such as incidence of heart failure, exercise tolerance, or need for cardiac rehabilitation. Some evidence suggests that reduced D2B time is associated with increased false positives,^23^ although we did not observe any false-positive CCL activations in this study.

## CONCLUSION

In this small, single-center, before-and-after study, we demonstrated that implementing push notifications to alert ED staff to prehospital ECG reception correlated with a small, but significant, reduction in ED delay of activating the CCL. Additionally, times to CCL activation were more consistent. We did not observe a significant change in the proportion of cases that received pre-arrival activation. While future research is needed to determine the clinical significance, it is possible that push notifications have a role in optimizing ED workflow for prehospital STEMI patients.

## Figures and Tables

**Table 1:** Patient demographics compared before and after implementation of push notification.

	Before (n=32)	After (n=13)	P value
Age			0.900
Range	39.17–88.93	34.70–85.84	
Median	65.20	66.90	
IQR	16.70	19.80	
Gender			0.586
Male	22	7	
Female	9	5	
Unknown	1	1	
Race			0.218
Asian	1	0	
Hispanic	1	3	
Other	1	0	
White	28	9	
Unknown	1	1	
Risk factors			
CAD	15	1	0.017
HTN	22	6	0.287
HLD	20	4	0.091
DM	7	2	1.000
Smoker	9	4	1.000
30-day mortality	5	0	0.303

*IQR*, interquartile range; *CAD*, coronary artery disease; *HTN*, hypertension; *HLD*, hyperlipidemia; *DM*, diabetes mellitus.

**Table 2:** Summary of emergency department (ED) delay before and after implementation of a push notification system.

	Before	After	Difference
Pre-arrival activation	n=14	n=6	
ED delay			
Median	6.27	3.00	3.27 (p=0.005)
IQR	4.58–15.43	2.50–4.50	
D2B			
Mean±std dev	57.00±15.20	48.67±12.80	8.33 (p=0.253)
Post-arrival activation	n=18	n=7	
ED delay			
Median	1.00	1.50	0.50 (p=0.553)
IQR	0.00–4.00	0.00–10.75	
D2B			
Mean±std dev	51.50±16.40	44.00±5.66	7.5 (p=0.137)

*IQR*, interquartile range; *std dev*, standard deviation; *D2B*, door-to-balloon time.

All units are decimal minutes.
